# Sourdough Fermentation Degrades Wheat Alpha-Amylase/Trypsin Inhibitor (ATI) and Reduces Pro-Inflammatory Activity

**DOI:** 10.3390/foods9070943

**Published:** 2020-07-16

**Authors:** Xin Huang, Detlef Schuppan, Luis E. Rojas Tovar, Victor F. Zevallos, Jussi Loponen, Michael Gänzle

**Affiliations:** 1 Department of Food and Nutrition, Faculty of Agriculture and Forestry, University of Helsinki, PL 66, FI-00014 Helsinki, Finland; 2 Fazer lab, Fazer Group, 01230 Vantaa, Finland; jussi.loponen@fazer.com; 3 Institute of Translational Immunology and Research Center for Immunotherapy, University Medical Center, Johannes Gutenberg University, 55131 Mainz, Germany; detlef.schuppan@unimedizin-mainz.de (D.S.); victor.zevallos@northumbria.ac.uk (V.F.Z.); 4 Division of Gastroenterology, Beth Israel Deaconess Medical Center, Harvard Medical School, Boston, MA 02215, USA; 5 Department of Agricultural, Food and Nutritional Science, University of Alberta, Edmonton, AB T6G 2P5, Canada; rojastov@ualberta.ca (L.E.R.T.); mgaenzle@ualberta.ca (M.G.); 6 Nutrition and Food Research Group, Department of Applied and Health Sciences, University of Northumbria, Newcastle Upon Tyne NE1 8ST, UK; 7 College of Bioengineering and Food Science, Hubei University of Technology, Wuhan 430068, China

**Keywords:** wheat sensitivity, bioactivity, innate immunity, fermentation, lactic acid bacteria

## Abstract

The ingestion of gluten-containing foods can cause wheat-related disorders in up to 15% of wheat consuming populations. Besides the role of gluten, α-amylase/trypsin inhibitors (ATI) have recently been identified as inducers of an innate immune response via toll-like receptor 4 in celiac disease and non-celiac wheat sensitivity. ATI are involved in plant self-defense against insects and possibly in grain development. Notably, they are largely resistant to gastrointestinal proteases and heat, and their inflammatory activity affects not only the intestine, but also peripheral organs. The aim of this study was to understand the changes of ATI throughout the sourdough and yeast-fermented bread-making processes. ATI tetramers were isolated, fluorescein-labelled, and added to a mini-dough bread-making system. When the pH decreased below 4.0 in sourdough fermentation, the ATI tetramers were degraded due to the activation of aspartic proteases, whilst in yeast fermentation, ATI tetramers remained intact. The amylase inhibitory activity after sourdough fermentation decreased significantly, while the concentration of free thiol groups increased. The glutathione reductase activity of *Fructilactobacillus sanfranciscensis* did not contribute to the reduction of ATI tetramers. Compared to the unfermented wheat, sourdough fermentation was able to decrease the release of pro-inflammatory cytokines monocyte chemoattractant protein-1 (MCP-1) and tumor necrosis factor alpha (TNF-α) in quantitative ATI extracts added to the human monocytic cell line THP-1. The current data suggest that sourdough fermentation can degrade ATI structure and bioactivity, and point to strategies to improve product development for wheat sensitivity patients.

## 1. Introduction

Wheat is a major staple food for human consumption, and wheat ingredients have been widely used for processed foods. However, an increasing number of people suffer from wheat-related disorders. Of these wheat related disorders, non-celiac wheat sensitivity (NCWS) is a heterogeneous and poorly defined condition that is diagnosed by exclusion of other wheat-induced immunological disorders, including allergies, wheat-dependent exercise-induced anaphylaxis, and autoimmune diseases such as celiac disease, gluten ataxia, and dermatitis herpetiformis [1,2,3,4,5]. NCWS is manifested by intestinal and multiple extra-intestinal complaints, while standard endoscopic workup and routine laboratory parameters are usually normal [3]. Intestinal and extra-intestinal symptoms of NCWS can overlap with celiac disease (CD) and irritable bowel syndrome (IBS) [6,7]. CD affects about 1% of most human populations, has a strong genetic component, and is caused by an adaptive, i.e., T-cell-mediated, immune response to defined gluten peptides after their modification by the autoantigen tissue transglutaminase [8,9,10].

Intestinal symptoms of NCWS are not triggered by gluten proteins but relate to wheat fructans, which protect the plant in cold and drought conditions, as well as wheat proteins including amylase trypsin inhibitors that protect the wheat grain against herbivores and fungal attack [11,12]. A pro-inflammatory effect is best documented for α-amylase/trypsin inhibitors (ATIs) that activate the toll-like receptor 4 (TLR4) complex on myeloid cells, monocytes, macrophages, and dendritic cells, to induce a mild intestinal inflammation, disturb tight junctional integrity, and promote extra-intestinal diseases like allergies, the metabolic syndrome, and fatty liver disease [13,14,15,16,17,18]. ATI are also prominent allergens in immediate-type, immunoglobulin E (IgE)-Antibody-positive allergies, such as bakers’ asthma [1]. Moreover, atypical IgE and skin test-negative wheat allergies are a prominent cause of IBS [4,5]. Therefore, ATI are considered important contributors to CD and NCWS but also other intestinal and extra-intestinal immune disorders [2,7,19,20].

ATI are present in many plants and play a protective role by inhibition of α-amylases from insects and mites [21]. ATI are associated with storage proteins in endosperm and are involved in grain maturation [22]. In bread wheat, ATI represent up to 4% of total wheat proteins, and consist of at least 14 types of subunit proteins [23]. The nomenclature of ATI subunits is inconsistent. They include wheat ATI monomer with molecular weight (MW) 12 kDa, often referred to as 0.28 inhibitor based on its electrophoretic mobility, ATI homodimer with MW 24 kDa, referred to as 0.19 and 0.53 inhibitors, and ATI heterotetramers with MW 60 kDa, also referred to as CM proteins. CM proteins, termed for their solubility in chloroform:methanol mixtures, include subunits CM1, CM2, CM3, CM16, and CM17 [23]. Each ATI subunit ranges from about 11 to 16 kDa in MW and contains 10 cysteine residues (except ATI 0.53 with 9 cysteine residues) that form five intramolecular disulfide bonds while dimers and tetramers associate in a non-covalent manner [23]. The multimeric forms of ATI determine their biological activity and the ATI tetramer is five times more active than the monomer [14]. In addition, the compact, highly disulfide-linked secondary structure of ATI is needed for their biological activity, i.e., the activation of TLR4 [13]. Reduction of these disulfide bonds or hydrolysis of ATI by proteinase K significantly decreased their bioactivity [13]. Primary structures are also involved, since 2 peptides were identified that inhibited the interaction of CM3 with TLR4 [24]. CM3 and 0.19 are the most prevalent ATI species, and were shown to have comparable bioactivity when expressed recombinantly in eukaryotic cells [13]. ATI of gluten-containing grains (wheat, barley, and rye) have potent inflammatory activity, while structurally less related ATI of oats, maize, rice, and legumes have no or low activity [14].

Food processing alters the conformation of allergen proteins and may abolish, and in rarer cases, increase their allergenicity [25]. In one study, IgE-antibodies of wheat-allergic patients reacted with in vitro digested bread crumb and crust, but not with unheated bread dough [26]. The TLR4-stimulating bioactivity of ATI is reduced by 20–50% after bread- and biscuit-making, compared to unprocessed wheat flour [14]. Malting and germination activates cereal endogenous proteolytic enzymes and induces the degradation of gluten proteins and celiac-toxic gluten epitopes [27,28,29]. Sourdough fermentation also alters the composition and activity of wheat proteins, since it reduces the pH to the optimum pH of endogenous cereal proteases and thus stimulates proteolysis [28,30]. In addition, heterofermentative lactobacilli in sourdough accumulate glutathione, resulting in the reduction of intramolecular and intermolecular disulfide bridges in wheat gluten and other proteins [28,31]. Sourdough fermentation is widely used in artisanal and industrial bread production [32], however, the changes of the immunologically highly relevant ATI during production of sourdough bread remains undefined.

The aim of this study was to investigate the wheat ATI tetramers in wheat sourdough- and yeast-fermentation bread-making. This study provides fundamental knowledge on the molecular changes of ATI tetramer due to fermentation, proteolytic, and heat treatment during these two bread-making processes. The fate of ATI was assessed by a three-pronged approach including (i) the analysis of dough proteins by sodium dodecyl sulfate–polyacrylamide gel electrophoresis (SDS-PAGE) and assessment of the inhibition of amylases by these proteins, (ii) the assessment of fluorescein-labelled ATI tetramer in sourdough fermentation and a model bread-making protocol, and (iii) the assessment of the inflammatory activity of sourdough extracts. To assess the role of cereal aspartic proteases and the microbial accumulation of low molecular weight thiols, sourdough fermentations were carried out in presence of protease inhibitors, and with isogenic strains of *Fructilactobacillus sanfranciscensis* that differ with respect to glutathione reductase activity.

## 2. Materials and Methods

### 2.1. Sourdough and Yeast Dough Making

Pastry wheat flour (hexaploid endosperm white flour) with no ascorbic acid (protein content 12.5%) and rye sourdough starter were supplied from Fazer Mills, Lahti, Finland. Fermaid SSR inactive yeast containing 2.5–3% glutathione (GSH) was obtained from Lallemand, Finland. Fresh yeast was purchased from Suomen Hiiva Oy, Lahti, Finland. Dough yield for both sourdough and yeast dough was 200. Doughs 1, 2, and 3 contained 250 g flour, 250 g water, and 5 g fresh yeast. Doughs 4 and 5 contained 250 g flour, 250 g water, 5 g GSH-containing inactive yeast, and 5 g fresh yeast. Dough 6, as a chemical-acidified dough control, contained 250 g flour and 250 mL 200 mM Na Acetate at pH 3.5. Dough 7, wheat sourdough, contained 250 g flour, 250 g water, and 15 g rye sourdough starter. Dough 8 consisted of 250 g flour, 250 mL 200 mM Na Acetate, pH 3.5, and 5 g inactive yeast. The mixing time was 5 min with a HOBART dough mixer. The fermentation time and temperature for each dough was: Dough 1, 30 °C, 1 h; Dough 2, 30 °C, 17 h; Dough 3, 4 °C, 17 h; Dough 4, 30 °C, 1 h; Dough 5, 30 °C, 17 h; Dough 6, 30 °C, 17 h; Dough 7, 30 °C, 17 h; Dough 8, 30 °C, 17 h. The pH of each dough at the end of the fermentation was measured with a Lab pH meter PHM 92 (Radiometer Copenhagen).

### 2.2. SDS-PAGE

The yeast doughs and sourdoughs were lyophilized and grounded in a mortar. An aliquot of 2 g dried dough flour was extracted with 150 mM NaCl at ambient temperature to separate the albumin/globulin fraction of the dough flour. The protein solution was also heated at 60 °C for 30 min to inactivate beta-amylases and then the supernatant was dialyzed against water to remove the salt by a membrane cut-off of 3.5 kDa. Followed by lyophilization, the albumin/globulin fraction of dough flour before and after heat treatment were analyzed by SDS-PAGE. Wheat flour as control was treated as described before. The dried samples were mixed with 4× lithium dodecyl sulfate (LDS) sample buffer and 10× sample reducing agent and boiled for 3 min and separated on NuPage Bis-Tris 10% gel with MES (2-(N-morpholino)ethanesulfonic acid) SDS running buffer (Invitrogen, Thermo Fisher Scientific, Waltham, MA, USA). Novex Sharp pre-stained protein standard (LC5800) or SeeBlue Plus2 Pre-stained standard (LC5925, Thermo Fisher Scientific) served as molecular markers (myosin 198 kDa, phosphorylase 98 kDa, bovine serum albumin 62 kDa, glutamic dehydrogenase 49 kDa, alcohol dehydrogenase 38 kDa, carbonic anhydrase 28 kDa, myoglobulin 17 kDa, lysozyme 14 kDa, and aprotinin 6 kDa). Running condition was 200 V for 35 min. The gel was stained by Commaasie Brilliant Blue R-250.

### 2.3. Amylase Inhibitory Activity

A 100 mg aliquot of the albumin/globulin fraction of dough flour was dissolved in 1 mL 150 mM NaCl, and the protein concentration was measured by the Lowry method (Detergent compatible *DC* Protein assay, BioRad), and adjusted to the same concentration for each dough. The amylase inhibitory activity of the dough albumin/globulin fraction was measured using soluble potato starch (Sigma S2630) and α-amylase from porcine pancreas (PPA) (Sigma A3176) or crude α-amylase extract from the yellow mealworm *Tenebrio molitor* (TMA). For PPA, the reaction was conducted in the presence of 50 mM sodium phosphate, 50 mM NaCl, 0.5 mM CaCl_2_, 0.1% BSA, and pH 6.9, while for TMA it was performed in 20 mM Na-acetate, 100 mM NaCl, 1 mM CaCl_2_, 0.1% BSA, and pH 5.4 at ambient temperature [33]. A control sample was only incubated with the enzyme. The release of the maltose was measured spectrophotometrically at 540 nm, with a series of maltose solution standards, after reaction with 3,5-dinitrosalicylic acid. Inhibition percentage of each dough was counted as =(Control_maltose_ − Dough_maltose_)/Control_maltose_ × 100%. Each assay was performed in triplicates.

### 2.4. Determination of Free Thiols

The albumin/globulin fraction of 9 mg freeze-dried dough flour was completely dissolved in 1 mL of 5% sodium dodecyl sulfate (SDS), 0.1 M Tris-HCl, pH 8.0, buffer at 60 °C for one hour. 100 µL of the solution was diluted with 900 µL 0.1 M Tris-HCl, pH 8.0, and mixed with 50 µL 10 mM DTNB (Ellman’s reagent, 5,5-dithio-bis-(2-nitrobenzoic acid)). Absorbance at 412 nm before and after adding DTNB was measured. Protein concentration was determined at 280 nm. The thiol content was calculated using molar extinction coefficient Δε412 = 14,150 M^−1^ cm^−1^, and expressed as µM/mg protein. Each assay was performed in triplicates.

### 2.5. Isolation and Characterization of ATI

Wheat flour was defatted with methanol:diethyl ether (1:1, v/v) and extracted with 150 mM NaCl (m/v) at a 1:5 ratio overnight at 4 °C. After centrifugation at 15,000× *g*, the supernatant was collected and proteins were precipitated by 20% ammonium sulfate at ambient temperature. The precipitated proteins were dissolved in 100 mM ammonium acetate, pH 6.9, and dialyzed against the same buffer overnight. The protein solution was cleared by centrifugation and heat-treated at 70 °C for one hour to denature all β-amylases. After centrifugation, the crude extract was loaded on a size-exclusion column with Sephadex G-100 (100 cm × 26 mm) connected to an Äktä prime plus system (GE Healthcare, Uppsala, Sweden), and eluted with the same acetate buffer (flow rate 0.5 mL/min). Fractions with molecular size around 60 kDa were collected according to the marker BSA (66 kDa). The fractions were combined, dialyzed, and lyophilized for the study of ATI tetramers. For identification, the amylase inhibitory activity of the collected fractions against α-amylase from porcine pancreas (PPA) and α-amylase from *Tenebrio molitor* (TMA) was confirmed. The SDS-PAGE gel bands at Mr 15 kDa were excised for mass spectrometry MS identification. In short, the proteins were reduced and alkylated and in-gel digested with sequencing grade trypsin (Promega, Madison, WI, USA) overnight at 37 °C. The tryptic peptides were identified by matrix-assisted laser desorption ionization tandem time-of-flight (MALDI-TOF/TOF) (UltrafleXtreme 2000 Hz, Bruker Daltonics, Bremen, Germany) using a matrix solution of α-cyano-4-hydroxy cinnamic acid (Sigma, USA). The identified peptides were searched against the SwissProt sequences from *Triticum aestivum* using Matrix Science’s Mascot Daemon (Matrix Science Ltd. London, UK). The searching parameters were 0.1 Da precursor tolerance, 1 Da MS/MS fragment tolerance, fixed modification of carbamidomethylation of cysteine, and variable modification of oxidation of methionine, and one trypsin miscleavage was allowed. Proteins were identified by their unique peptides [34].

### 2.6. Sourdough and Yeast Bread-Making with Fluorescein-Labelled ATI

Isolated ATI tetramer at 20 mg/mL in 100 mM pH = 9.0 sodium carbonate-bicarbonate buffer was reacted with 10 mg/mL fluorescein isothiocyanate (FITC) in dimethyl sulfoxide (DMSO) for two hours in the dark. The excess FITC was removed by gel filtration on a PD-10 column and FITC-labelled ATI tetramer was eluted with 50 mM sodium phosphate, pH 6.9. Purified FITC-labelled ATI solution was added to mini-dough systems to follow its changes during bread-making. Type I sourdough bread includes two stages of fermentation. The first stage is long time sourdough fermentation mainly with lactobacilli, and the second stage is bread dough fermentation with the addition of wheat flour and baker’s yeast. Yeast bread is only one stage fermentation with direct addition of baker’s yeast. The mini-sourdough was prepared by mixing 1.5 g pastry white wheat flour (hexaploid modern) with 1.5 g water plus the inoculums or rye sourdough starter (5% m/m), and fermented for 24 h at 30 °C. For the bread dough, an additional 3.5 g of wheat flour was added together with 1.5 g water, including 1% (m/m) dried yeast (Active dry yeast, Fleischmann’s, Mississauga, ON, Canada), 2% (m/m) salt, and 2% (m/m) sugar. After yeast fermentation for 2.5 h at 30 °C, the bread dough was placed in a boiling water bath for 8 min until the dough temperature reached 95 °C. The labelled ATI was dosed as 1% of wheat flour protein at both the sourdough and bread dough stage, respectively. Samples were taken for analysis of labelled ATI at the sourdough stage (A): A1: after sourdough mixing as control, A2: after lactobacilli fermentation, A3: after yeast fermentation, and A4: after baking. At the bread dough stage (B): B1: after bread dough mixing, B2: after yeast fermentation, and B3: after baking. Yeast bread was prepared with 5 g wheat flour, 3 g water, 1% (m/m) dried yeast, 6 mg labelled ATI (1% of wheat flour protein), and fermented at 30 °C for 2.5 h (wheat fermentation stages, Y): Y1: after dough mixing, Y2: after yeast fermentation, and Y3: after baking. After sourdough fermentation, pH and cell counts were analyzed immediately.

The inoculum *Fructilactobacillus sanfranciscensis* DSM20451^T^ (previously: *Lactobacillus sanfranciscensis*) [35] were cultivated in modified MRS (De Man, Rogosa and Sharpe) agar with 10 g/L maltose and 5 g/L fructose anaerobically at 30 °C, and mutant *F. sanfranciscensis* DSM20451^T^ *ΔgshR*, without glutathione reductase activity [31], were cultivated with an additional 10 mg/L erythromycin. The rye sourdough starter consisted of lactic acid bacteria in 9 log colony-forming units (CFU) g^−1^ including *F. sanfranciscensis*, *Lactobacillus crispatus*, *Weissella cibaria*, and yeast *Kazachstania humilis* in 7 log CFU g^−1^. Chemically acidified doughs were prepared by mixing 1.5 g wheat flour and 1.5 g solution mixture of lactic acid and acetic acid at pH 4.0 and pH 5.0, respectively. Bacterial cell counts were monitored by plating appropriate dilution of sourdough samples with mMRS agar and incubated anaerobically at 30 °C for 2–3 days using the Whitley automatic spiral plater (Whitley Scientific, Bingley, UK) [31].

### 2.7. Confirmation of Single Strain Fermentation by qPCR (Quantitative Polymerase Chain Reaction)

The identity of the strains *F. sanfranciscensis* DSM20451^T^, and *F. sanfranciscensis* DSM20451^T^ *ΔgshR* was confirmed by high-resolution melting quantitative PCR (HRM-qPCR) following the conditions described by Lin and Gänzle [36]. In short, 30 mg of freeze-dried sourdough sample were mixed with 2.5 mL of sterile 0.8% (*w*/*v*) NaCl solution. The mixture was centrifuged at 5000× *g* for 5 min and supernatant was collected. The supernatant was centrifuged at 5000× *g* for 15 min to harvest the cells. DNA was extracted using a DNeasy Blood and Tissue Kit (Qiagen, Germantown, MD, USA). For the PCR, a Rotor-Gene Q (Qiagen, Germantown, MD, USA) and Type-it HRM PCR Kit (Qiagen, Qiagen, Germantown, MD, USA) were used. The primers for the Experiment were Universal Primers targeting 16S (forward, 5′-TCC TAC GGG AGG CAG CAG T-3′; reverse, 5′-GGA CTA CCA GGG TAT CTA ATC CTG TT-3′). The conditions for the HRM-qPCR were denaturation for 5 min at 95 °C, then 45 cycles of denaturation at 95 °C for 10 s, annealing at 60 °C for 30 s, and extension at 72 °C for 10 s. For the final stage, the temperature was raised from 65 °C to 90 °C at 0.1 °C increments for 2 s at each step. The software used to process the data was PeakFit Software (Systat Software Inc., San Jose, CA, USA).

### 2.8. Analytical Size-Exclusion Chromatography

The samples collected from the mini-dough systems were lyophilized and ground in a mortar. The proteins were extracted from a 100 mg dried flour sample with 1 mL of 100 mM ammonium acetate, pH 6.9, filtered through a 0.45 µm pore membrane, and separated on a Superdex Peptide 10/300 GL column (GE Healthcare, Life Sciences) coupled to an Agilent 1200 high-performance liquid chromatography (HPLC) system with a diode array detector and a fluorescence detector (excitation = 488 nm, emission = 530 nm) at a flow rate of 0.5 mL/min in 100 mM ammonium acetate, pH 6.9, at ambient temperature. BSA (66 kDa), lysozome (14 kDa), and GSH (307 Da) served as standards. The ATI tetramer and monomer were identified by elution volume and fluorescence detection. The peak height ratio (tetramer:tetramer + monomer) was calculated to demonstrate ATI tetramer dissociation/degradation.

### 2.9. Sourdough Fermentation in the Presence of Protease Inhibitors

The sourdough contained 250 g flour, 250 g water, and 15 g rye sourdough starter in the presence of 20 µM aspartic protease inhibitor Pepstatin-A or 10 µM cysteine protease inhibitor E-64, (1*S*,2*S*)-2-(((*S*)-1-((4-Guanidinobutyl)amino)-4-methyl-1-oxopentan-2-yl)carbamoyl)cyclopropanecarboxylic acid, respectively. After lyophilization, the albumin/globulin extract of the dough flour was heat-treated and analyzed as in Section 2.2 by SDS-PAGE. To investigate the proteases effect on ATI tetramer, the same concentration of Pepstatin-A or E-64 was added to the mini-dough systems respectively, where fluorescein-labelled ATI tetramer was included. The fermentation was carried out for 17 h at 30 °C and the dough pH was measured at the end of the fermentation. The ATI tetramer was then analyzed by size-exclusion chromatography coupled with a fluorescence detector, as described in Section 2.8.

### 2.10. Bioactivity of Sourdough

Whole grain wheat flour was fermented with *F. sanfranciscensis* DSM20451^T^, *F. sanfranciscensis* DSM20451^T^ *ΔgshR*, *Limosilactobacillus reuteri* LTH5448 (previously *Lactobacillus reuteri*), or *Latilactobacillus sakei* LS8 (previously *Lactobacillus sakei*) [35], as described above, and the reaction was terminated by lyophillization. ATI (TLR4-stimulating) bioactivity was determined as described before [14,15]. Briefly, 1 g of fermented and lyophilized sourdough samples was sieved and defatted with 10 times (*w*/*v*) methanol/diethyl ether 1:1 (*v*/*v*) for 2 h at ambient temperature with constant stirring, followed by centrifugation at 2500× *g* for 20 min. After drying, the pellet was extracted with 5 volumes (*w*/*v*) 50 mM ammonium bicarbonate, pH 7.8, overnight at 4 °C, and the supernatant was saved. A second consecutive extraction under the same conditions was performed, which contributed another 20% of total extractable ATI, equivalent to 90–95% of total extractable ATI activity [14]. Protein content was measured using the bicinchoninic acid assay (Thermo Fisher Scientific, Bonn, Germany). The two extractions were combined, diluted in phosphate-buffered saline (PBS) or cell culture medium, and extracts with 10–250 µg protein/mL were added to mycoplasma-free cultures of ATI-reactive (TLR4-bearing) human THP-1 monocytes cells (TIB-202, ATCC, Wesel, Germany), seeded at densities of 2 × 105 to 1 × 106/mL in 0.2 to 1 mL medium in 96- or 24-well culture plates. An extract of a standard commercial wheat flour, bacterial lipopolysaccharide (LPS), or a blank control served as positive and negative controls, respectively. After a 0.5–16 h incubation, chemokines and cytokines released into the medium were quantified with validated enzyme-linked immunosorbent assays (ELISAs) for CCL2/MCP-1 and TNF-α (BD, Heidelberg; eBioscience, Frankfurt, Germany; R&D systems, Minneapolis, MN, USA).

### 2.11. Statistical Analysis

The significance test of amylase inhibition activity and free thiol groups results were conducted by SPSS 10.0, using one-way analysis of variance (ANOVA) and Tukey’s honest significance test (HSD) test (*p* < 0.05). Bioactivity statistical analysis was performed by one-way ANOVA multivariate analysis followed by Dunnett’s post-hoc test using Graph-Pad Prism v.6.0. *p* < 0.05 was considered significant.

## 3. Results

### 3.1. Changes of the Albumin/Globulin Fraction after Sourdough and Yeast Fermentation

**Figure 1:**
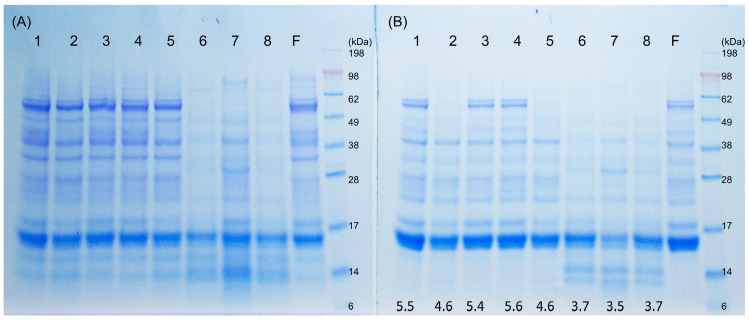
Sodium dodecyl sulfate–polyacrylamide gel electrophoresis (SDS-PAGE) under reducing condition of (**A**) the albumin/globulin extract of yeast-fermented doughs and sourdoughs, and (**B**) albumin/globulin extract of yeast-fermented doughs and sourdoughs after heat treatment. The pH value after fermentation is indicated below. Dough 1, yeast-fermented dough at 30 °C for 1 h; 2, yeast-fermented dough at 30 °C for 17 h; 3, yeast-fermented dough at 4 °C for 17 h; 4, yeast-fermented dough with glutathione at 30 °C for 1 h; 5, yeast-fermented dough with glutathione at 30 °C for 17 h; 6, chemically acidified dough, pH 3.5, at 30 °C for 17 h; 7, wheat sourdough at 30 °C for 17 h; 8, chemically acidified dough, pH 3.5, with glutathione at 30 °C for 17 h; F, wheat flour. The α-amylase/trypsin inhibitor (ATI) proteins migrate in the range of molecular weight 12–15 kDa.

**Figure 2:**
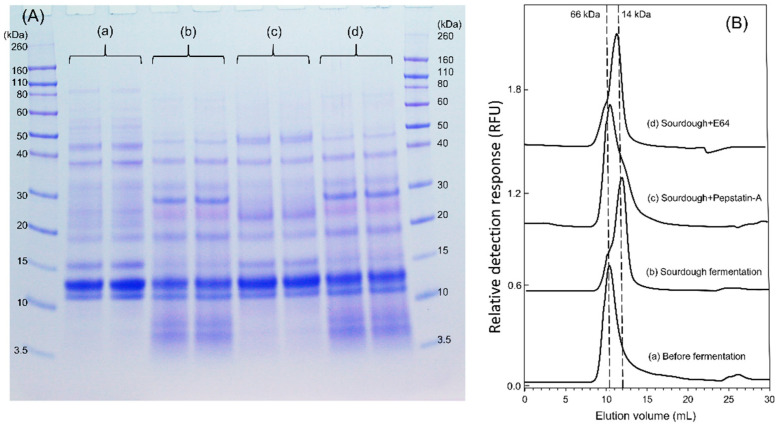
Effect of protease inhibitors on the wheat sourdough albumin/globulin extract and fluorescence-labelled ATI tetramer. (**A**) SDS-PAGE of albumin/globulin extract. (a) control before fermentation, (b) rye starter-wheat sourdough, pH was 3.6 after 17 h fermentation, (c) rye starter-wheat sourdough in the presence of 20 µM aspartic proteinase inhibitor Pepstatin-A, pH was 3.7 after 17 h fermentation, (d) rye starter-wheat sourdough in the presence of 10 µM cysteine proteinase inhibitor E-64, pH was 3.6 after 17 h fermentation. The replicates were from two individual fermentation. (**B**) Size-exclusion chromatogram of fluorescein isothiocyanate (FITC)-labelled ATI tetramer after sourdough fermentation in the presence of protease inhibitors. The samples were treated the same as in (**A**). Fluorescent detector was set at excitation 488 nm and emission 530 nm. Elution volume of molecular marker bovine serum albumin (66 kDa) and lysozyme (14 kDa) was indicated in broken lines.

The electrophoretic patterns characterize the composition of the albumin/globulin fractions before, during, and after sourdough and yeast fermentation (Figure 1). In general, sourdough 6, 7, and 8, with a final pH 3.5–3.7, showed a more intensive hydrolysis, and β-amylases with a molecular size around 60 kDa were degraded after sourdough fermentation, while they remained present after conventional flour and yeast fermentation (Figure 1A). After heat denaturation, yeast dough 2 and 5, extracted at a lower pH of 4.6, lead to precipitation of most β-amylases compared to yeast dough 1, 3, and 4, with the higher pH of 5.5, 5.4, and 5.6. Dough 1, 3, and 4 showed the same gel band patterns as native flour. In sourdough 6, 7, and 8, increased protein products of low molecular weight around 14 kDa were present (Figure 1B). Proteolysis in chemically acidified doughs and in sourdoughs has been attributed to gluten-associated aspartic proteases [28]; therefore, sourdough fermentation was also conducted in the presence of Pepstatin-A, an inhibitor of aspartic proteases, and in the presence of E64, an inhibitor of cysteine peptidases (Figure 2A). Proteolysis in sourdoughs was inhibited by Pepstatin-A but not by E-64, confirming the pH-dependent role of aspartic proteases on protein modifications (Figure 2A).

### 3.2. Amylase Inhibitory Activity of Dough Albumin/Globulin Extracts

**Figure 3:**
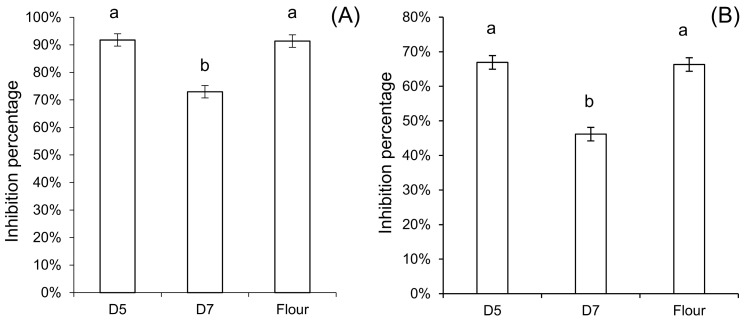
Inhibitory activity of the albumin/globulin extract after heat treatment against α-amylase from (**A**) yellow mealworm *Tenebrio molitor*, and (**B**) from porcine pancreas. D5, yeast-fermented dough; D7, wheat sourdough. Each letter indicates groups with statistical difference (*p* < 0.05, a vs. b) using triplicates and one-way analysis of variance (ANOVA) with Tukey’s honest significance test (HSD).

β-Amylases were first precipitated before comparing the amylase inhibitory activity of albumin/globulin extracts of sourdough and yeast-fermented dough. The inhibitory activity of yeast-fermented dough showed the same level as in the original flour, both against α-amylase from porcine pancreas (PPA) and *Tenebrio molitor* (TMA). However, the inhibitory activity of sourdough showed a significantly lower inhibitory activity than yeast-fermented dough and native flour in both the PPA and TMA assays (Figure 3).

### 3.3. Free Thiol Measurement of Dough Albumin/Globulin Extracts

**Figure 4:**
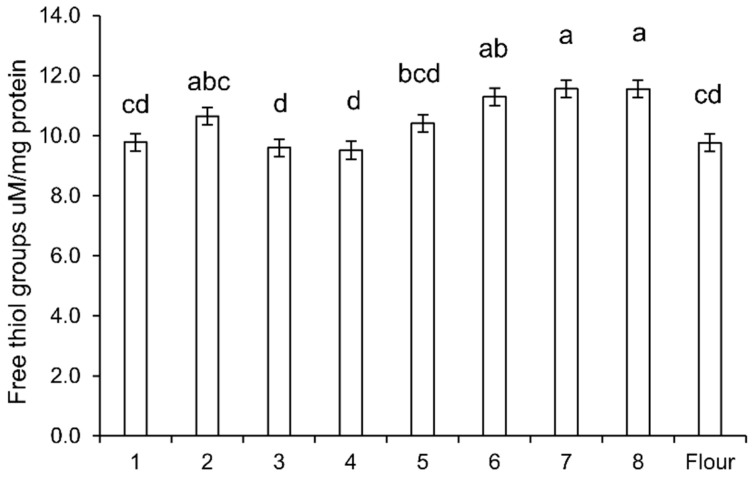
Free thiol groups in the albumin/globulin extracts of doughs. The number indicates the dough systems described in Figure 1. Dough 1, yeast-fermented dough at 30 °C for 1 h; 2, yeast-fermented dough at 30 °C for 17 h; 3, yeast-fermented dough at 4 °C for 17 h; 4, yeast-fermented dough with glutathione at 30 °C for 1 h; 5, yeast-fermented dough with glutathione at 30 °C for 17 h; 6, chemically acidified dough, pH 3.5, at 30 °C for 17 h; 7, wheat sourdough at 30 °C for 17 h; 8, chemically acidified dough, pH 3.5, with glutathione at 30 °C for 17 h; F, wheat flour. Bars that do not share a common superscript differ significantly (*p* < 0.05, one-way ANOVA with Tukey’s honest significance test (HSD) analysis).

The free thiol concentration of the dough albumin/globulin extracts correlated with dough pH (Figure 4). Short-fermented yeast dough had the same level of free thiols as the original wheat flour. With the lower pH in doughs 2 and 5, the free thiol groups slightly increased. Sourdough fermentation systems 6, 7, and 8 produced the highest level of free thiols that were significantly higher than in short-time yeast-fermented doughs. There was no significant difference between chemically acidified dough and rye starter wheat sourdough.

### 3.4. Characterization and Identification of ATI

**Figure 5:**
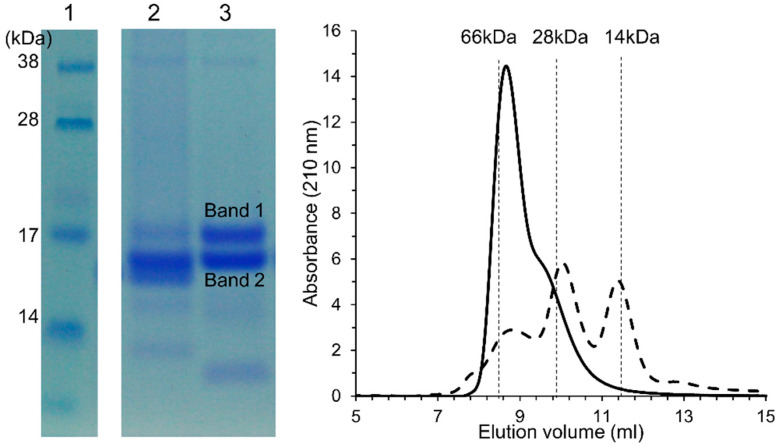
Identification of isolated ATI tetramers. SDS-PAGE of ATI tetramer isolates in the presence of 2% (*m*/*v*) dithiothreitol. (1) Molecular weight standard, (2) Commercial Sigma ATI control, containing mainly monomeric and dimeric ATI 0.19, 0.28, and 0.53 [14], and (3) ATI tetramer isolate. Size-exclusion chromatogram of ATI tetramer isolate (line) and Sigma ATI (broken line). Elution volume of molecular standard bovine serum albumin 66 kDa (8.6 mL), chymotrypsinogen 25.6 kDa (9.9 mL), and lysozyme 14 kDa (11.4 mL). As determined by mass spectrometry, Band 1 consists of the ATI tetramer CM3 subunit and Band 2 of the ATI tetramer CM2 subunit.

ATI were quantitatively extracted and tetramers of molecular weight around 60 kDa were characterized by size exclusion chromatograph and tandem mass spectrometry (MS/MS). Heat treatment denatured β-amylases, while inhibitory activity of ATI was retained both against PPA and TMA (data not shown). In SDS-PAGE, non-covalently assembled ATI tetramers were de-assembled to monomers by SDS-PAGE in reducing condition. In MS/MS analysis, Band 1 of the 60 kDa tetrameric ATI-fraction consisted of the ATI CM3 subunit (UniProt P17314), with four unique peptides (Score 295, protein sequence coverage 33%), and Band 2 consisted of the ATI CM2 subunit (UniPro P16851), with one unique peptide (Score 115, protein sequence coverage 17%) (Supplementary Figure S1). Size exclusion chromatography demonstrated that the purified and lyophilized ATI tetramer could be reconstituted in native form with molecular weight 60 kDa (Figure 5).

### 3.5. Behavior of ATI in Mini-Dough Systems

To determine the influence of yeast and sourdough fermentation on the changes of the ATI in bread-making, tetrameric ATI were labelled with fluorescein isothiocyanate, and added to sourdoughs or bread dough. Yeast bread was produced with a straight standard dough process and type I sourdough bread was produced with two stages of fermentation. For yeast bread fermentation, the fluorescence-labelled ATI was added directly at the beginning of the yeast fermentation. For sourdough bread-making, fluorescence-labelled ATI was added at two stages respectively, the first stage at the beginning of sourdough fermentation, and the second stage at the bread dough yeast fermentation. Moreover, sourdoughs were fermented with a multi-strain sourdough obtained from a bakery, or with defined and isogenic strains of *F. sanfranciscensis* that differ only with respect to glutathione reductase activity. To confirm that the fermentation was performed by the same strain, the melting temperature of amplicons from DNA isolated from sourdoughs was compared to the melting temperature of amplicons from DNA from pure cultures. All melting curves consisted of a single peak matching the peak obtained from the reference culture, confirming that the fermentation microbiota were identical to the inoculum. In sourdoughs fermented with pure cultures, the bacterial cell count reached 8.4–8.7 log CFU g^−1^, and the pH dropped to 4.0; in the multiple-strain sourdough, the pH dropped to 3.6 and the cell count reached 9.6 log CFU g^−1^.

**Figure 6:**
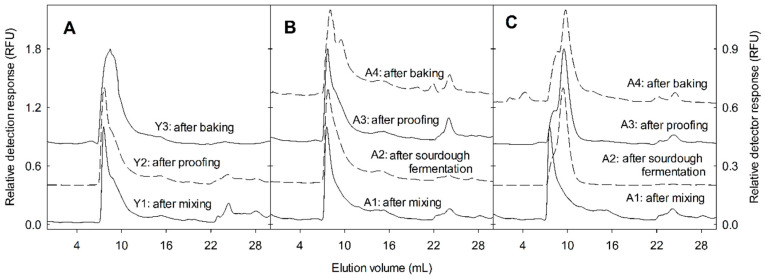
Size-exclusion chromatogram of fluorescein-labelled ATI in bread-making. Panel (**A**): Yeast-fermented bread, Panel (**B**): (III) chemically acidified dough at pH 5.0, Panel (**C**): (V) Rye starter-wheat sourdough. Fluorescence-labelled ATI was added to bread dough Panel (**A**) or to sourdough Panels (**B**,**C**). ATI was extracted after mixing of sourdough, after sourdough fermentation, or after mixing of bread dough, after proofing, and after baking. FITC-labelled ATI was separated by size exclusion chromatography coupled to a fluorescent detector set at excitation 488 nm and emission 530 nm. Chromatograms were normalized to the highest peak intensity in each chromatogram and were offset by 0.4 RFU. The elution volume of the molecular marker bovine serum albumin (66 kDa) was 7.25 mL, of lysozyme (14 kDa) was 9.86 mL, and of glutathione (307 Da) was 17.35 mL. The Roman number and the sample letters are the same as those indicated in Table 1.

The fate of ATI was initially monitored by size-exclusion chromatography, to identify dissociation of the tetramer to monomers, and proteolytic degradation. In yeast-fermented bread, fermentation had no effect on the molecular size of ATI tetramers, and only heat treatment induced a shift in the size distribution of ATI tetramers towards monomers (Figure 6A). In chemically acidified doughs acidified to pH 5.0, the ATI tetramers added stayed intact throughout sourdough fermentation (Figure 6B). When ATI was added to bread dough that also included chemically acidified dough incubated at pH 5.0, the yeast fermentation and heat treatment affected the distribution of ATI tetramers only slightly (Supplementary Figure S1B). In the wheat sourdoughs, fermentation drastically reduced ATI tetramers (Figure 6C). For the ATI dosed to bread dough that included rye starter-wheat sourdough, 36% of ATI tetramer was transformed to monomer during yeast fermentation, and heat treatment further reduced ATI (Supplementary Figure S2C). Sourdough fermentation in the presence of Pepstatin-A and E-64 indicated that the conversion of tetrameric ATI to smaller subunits was dependent on aspartic proteases (Figure 2B).

ATI disassembly was estimated by calculating the ratio of the ATI tetramers to ATI monomers in size exclusion chromatograms (Table 1). When FITC-labelled ATI was added to chemically acidified doughs, the disassembly was more extensive after incubation at pH 4.0 when compared to doughs incubated at pH 5.0 (Table 1). In doughs acidified to pH 4.0 as well as in wheat sourdough fermented with a multi-species culture, less than 30% of the ATI remained as tetramers. After mixing of the bread dough, proofing, and baking, less than 50% of the ATI remained as tetramers (Table 1). The disassembly of ATI tetramers was less extensive in sourdoughs fermented with pure strains of *F. sanfranciscensis,* and, notably, the expression of glutathione reductase did not affect ATI degradation (Table 1)**.** Addition of FITC-labelled ATI to bread dough that also included chemically acidified dough (acidified to pH 4.0 or 5.0), or sourdoughs fermented with pure or mixed strains of lactobacilli resulted in a less extensive degradation of the ATI tetramer, and approximately 60% of the ATI tetramers remained intact (Table 1). Disassembly of FITC-labelled ATI during proofing was observed only in bread dough prepared with chemically acidified dough (pH 4.0), or with multi-species sourdough (Table 1).

### 3.6. Inflammatory Bioactivity of Protein Extracts from Sourdoughs

**Figure 7:**
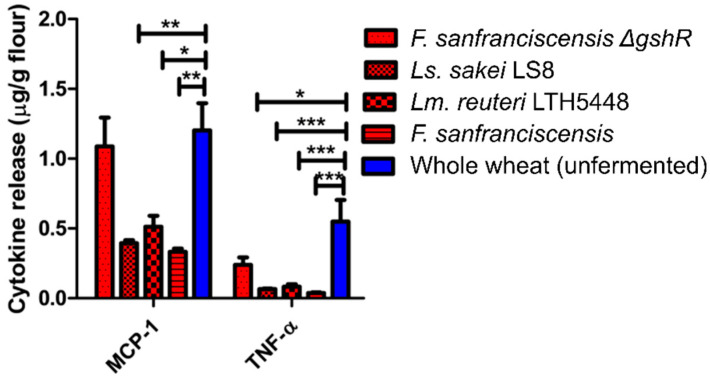
ATI bioactivity of whole wheat fermented by four Lactobacillus strains ATI were extracted from freeze-dried doughs, and extracts added to toll-like receptor 4 (TLR4) expressing THP-1 human monocytic cells. The release of monocyte chemoattractant protein-1 (MCP-1) and tumour necrosis factor alpha (TNF-α) into the culture medium was quantified by enzyme-linked immunosorbent assay (ELISA). (* *p* < 0.05; ** *p* < 0.01; *** *p* < 0.001).

To determine whether modifications of the ATI during sourdough fermentation also result in an altered biological activity, the release of three pro-inflammatory cytokines that are induced by the TLR4-stimulating activity of ATI were measured in quantitative ATI extracts of doughs fermented with *F. sanfranciscensis* and *F. sanfranciscensis ΔgshR* (Figure 7). Sourdoughs fermented with pure strains of *Ls. sakei* or *Lm. reuteri* were additionally included as controls (Figure 7). The induction of MCP-1 by standardized extracts from sourdoughs was significantly reduced if sourdoughs were fermented by wild-type strains of *F. sanfranciscensis*, *Ls. sakei*, or *Lm. reuteri*. Fermentation with *F. sanfranciscensis ΔgshR* did not reduce MCP-1 induction relative to the unfermented wheat control. The induction of TNF-α by extracts from sourdoughs was reduced in sourdoughs fermented by all strains, including *F. sanfranciscensis ΔgshR* (Figure 7).

## 4. Discussion

We studied the fate of wheat amylase trypsin inhibitors (ATI), specifically the prominent tetramer-forming ATI CM3 and CM2, throughout sourdough and yeast-fermented bread-making. The family of ATI proteins has recently become the focus of great concern, since wheat ATI have been identified as important pro-inflammatory proteins in wheat and related grains, with far-reaching consequences for human health [13,14,15,16,17,18]. We quantified ATI activity, biochemically characterized their disassembly/degradation, and measured their pro-inflammatory activity in differently fermented dough extracts, to show that disulfide-bond reduction and a pH-dependent proteolytic disassembly/degradation of ATI tetramers occurred with sourdough fermentation.

The SDS-PAGE protein patterns showed that disassembly/degradation of ATI on sourdoughs was more extensive when compared with yeast-fermented dough. ATI are water-soluble proteins and therefore, are classified as albumins. The degradation of albumins, including ATI, in sourdoughs has not been investigated before, as most previous studies have focused on the changes of gluten proteins during sourdough fermentation [27,28,37]. Some gluten-derived hydrolysis products appeared in the albumin/globulin fraction, but the hydrolysis products with bands smaller than 28 kDa that we focused on and that largely correspond to ATI were not recognized by polyclonal gliadin antibody (data not shown).

The decrease of amylase inhibitory activity further supported the modification of the tetrameric ATI-complex by sourdough fermentation. The increase of free thiols in sourdough suggested that reduction of disulfide bonds indeed occurred in sourdoughs. Reduction of disulfide bonds can decrease the inhibitory activity of ATI due to conformational alterations and unfolding, especially at higher temperature, but also due to the fact that reduction of disulfide bonds that opens up the compact protein structure makes the ATI more susceptible for proteolytic degradation [13].

Sourdough fermentation with heterofermentative lactobacilli generates reducing conditions through glutathione reductase activity of lactobacilli, which reduces oxidized glutathione (GSSH) to glutathione (GSH) [31]. The GSH and GSSH together undergo sulfhydryl/disulfide bond interchanges with other disulfide-containing proteins [38]. Glutathione reductase activity of *F. sanfranciscensis* reduced the concentration of ovotransferrin, an egg white allergen, in sourdough [39]. However, the present study provided no evidence that glutathione reductase contributes to the reduction of ATI tetramers. A possible reason is the production of extracellular GSH from the lactobacilli, since content of low-molecular weight thiols was unchanged after 5 h of fermentation, with a decrease only at the beginning of the fermentation (3.5 h) [40]. When comparing yeast fermentation and sourdough fermentation, there was no reduction of ATI in all yeast fermentation stages. It was shown before that yeast fermentation increased yeast intracellular GSH [41], which after baking heat treatment, was released. This may have affected the ATI, as a decrease in tetramers vs. monomers was observed in all samples after heating. However, here, we could not determine the state of intra-chain disulfide bonds of each ATI subunit by using size-exclusion chromatography.

The comparison of chemically acidified dough incubated at pH 4.0 or 5.0 with standard yeast dough demonstrated that the pH was a crucial factor to reduce ATI integrity. In the chemically acidified dough at pH 4.0, and in wheat sourdough (rye starter and single strains), monomer formation from ATI tetramers was observed. This process was more intensive in the chemically acidified control at pH 4.0 and in rye starter-wheat sourdough, which rapidly acidified to pH 3.6, when compared to fermentations with single strains of *F. sanfranciscensis*, which acidified to pH 4.0. In addition, sourdough fermentation in the presence of the aspartic protease inhibitor Pepstatin-A indicated that the pH-dependent enzyme activity of endogenous proteinases from the flour is instrumental [28]. Degradation of ATI CM proteins from barley was also observed when incubated with purified aspartic proteinases from resting and germinated barley seeds at pH 4.5 [42].

The role of sourdough fermentation in reduction and/or disassembly of bioactive ATI tetramers was additionally by assessment of cytokines in the monocytic cell line THP-1. ATI-bioactivity and their pro-inflammatory activity, which is finally responsible for adverse health effects [13,14,15,16,17,18], were significantly reduced in sourdough extracts when compared to wheat flour, further suggesting a significant inactivating effect of the sourdough fermentation on ATI bioactivity.

While the biochemical, analytical, and immunological experiments described in this study consistently describe conversion of bioactive ATI tetramers during sourdough baking, the specific contribution of this conversion to wheat tolerance in NCWS remains to be determined. First, the use of sourdough in bread production is highly variable. The most extensive fermentation is achieved in type I sourdough processes that use sourdough as the sole leavening agent, while many industrial processes that employ sourdough in conjunction with baker’s yeast use only a small proportion of microbially inactive sourdough [43]. Second, the conversion of ATI tetramer in sourdough bread-making is only partial in this study and ATI monomers retain biological activity, albeit strongly reduced [14]. Third, while the use of sourdough to improve tolerance of wheat in NCWS has been suggested in public and social media [44], it is poorly documented in the scientific literature. In a pilot study with IBS patients [32], sourdough bread that contained less ATI as determined by SDS-PAGE was compared to yeast-fermented bread in a clinical trial with IBS patients. Gastrointestinal symptoms after consumption of control bread or sourdough bread were not significantly different. In a randomized, double-blind, cross-over study, tolerance of pasta and bread fermented with lactobacilli and fungal proteases was compared but only one questionnaire showed a lower score for IBS patients [45]. In both studies [32,45], effective degradation or reduction of ATI were poorly documented and the study subjects were recruited from IBS patients. In IBS patients, effects of ATI degradation are confounded by reduced levels of fermentable oligo-, di-, mono-saccharides, and polyols (FODMAPs) in sourdough bread. FODMAPs contribute to adverse intestinal symptoms in a majority of IBS patients, and particularly, the study of Laatikainen et al. [32] used bread where FODMAPs were strongly reduced in comparison to the control. A small study with healthy volunteers documented that consumption of sourdough bread improved satiety and reduced adverse gastrointestinal symptoms when compared to bread produced with brewer’s yeast. Because sourdough bread also reduced breath hydrogen levels, reduced intestinal fermentation of FODMAPs likely contributed to this effect [46]. Even a moderate reduction of ATI in combination with reduced levels of FODMAPs and the potential reduction of other potentially offending wheat proteins in sourdough bread may thus increase tolerance of wheat bread in some or in many individuals with IBS or NCWS.

## 5. Conclusions

In conclusion, this work provided fundamental knowledge on the fate of pro-inflammatory wheat ATI in different strategies of bread-making. ATI tetramers were disassembled/degraded and showed a significantly lower pro-inflammatory bioactivity after sourdough fermentation. Sourdough was the main leavening agent for bread production until the late 19th century. The introduction of baker’s yeast in 1871 resulted in a gradual replacement of sourdough fermentation by a straight, short-term dough processes. The use of sourdough in bread production has increased again in the past decade, owing to the superior organoleptic properties of sourdough bread; this study, apart from prior studies related to the degradation of FODMAPs during sourdough fermentation [47], suggested that the product development strategy using sourdough fermentation may benefit the community by reducing the severity of inflammatory non-celiac wheat sensitivity.

## Figures and Tables

**Table 1:** The pH value and bacterial cell count after fermentation in the mini-dough system, and ATI tetramer degradation throughout the bread-making process. The degradation of ATI tetramers was expressed as the ratio of the peak height of the ATI tetramer divided by the peak height of the ATI monomer in size-exclusion chromatograms.

	Bread System	pH	CFU g^−1^ log10	A1	A2	A3	A4	B1	B2	B3
I	*Fructilactobacillus sanfranciscensis*	4.01 ± 0.05	8.37 ± 0.05	1	0.62	0.62	0.56	1	1	0.61
II	*Fructilactobacillus sanfranciscensis ΔgshR*	4.07 ± 0.04	8.71 ± 0.03	1	0.64	0.63	0.55	1	1	0.63
III	Control, pH 5.0	-	-	1	1	1	0.61	1	1	0.60
IV	Control, pH 4.0	-	-	1	0.26	0.38	0.42	1	0.63	0.61
V	Wheat sourdough	3.69 ± 0.04	9.57 ± 0.19	1	0.25	0.27	0.36	1	0.64	0.57

Note: For ATI dosed at the sourdough stage, A1: after sourdough mixing (control), A2: after lactobacilli fermentation, A3: after yeast fermentation, A4: after baking. For ATI dosed at the bread dough stage, B1: after bread dough mixing, B2: after yeast fermentation, B3: after baking. CFU: colony-forming unit.

## Supplementary Materials

The following are available online at https://www.mdpi.com/2304-8158/9/7/943/s1: Figure S1: Protein sequences of identified ATI tetramers. Unique peptides underlined were identified by MALDI-TOF/TOF, Figure S2: Size-exclusion chromatogram of fluorescence-labelled ATI in bread-making, Panel A: Yeast-fermented bread, Panel B: (III) chemically acidified dough at pH 5.0, Panel C: (V) Rye starter-wheat sourdough. FITC-labelled ATI was added to bread dough. ATI was extracted after mixing of sourdough, after sourdough fermentation, or after mixing of bread dough, after proofing, and after baking. FITC-ATI was separated by size exclusion chromatography coupled to a fluorescent detector set at excitation 488 nm and emission 530 nm. Chromatograms were normalized to the highest peak intensity in each chromatogram, and were offset by 0.4 RFU. The elution volume of the molecular marker bovine serum albumin (66 kDa) was 7.25 mL, of lysozyme (14 kDa) was 9.86 mL, and of glutathione (307 Da) was 17.35 mL. The Roman number and the sample letters are the same as those indicated in Table 1.
