# Supplementary material for: Sourdough Fermentation Degrades Wheat Alpha-Amylase/Trypsin Inhibitor (ATI) and Reduces Pro-Inflammatory Activity
Source: Foods. 2020 Jul 16;9(7):943. doi: 10.3390/foods9070943 (PMC7404469; doi:10.3390/foods9070943)
Supplement: Supplementary file 1 [file foods-09-00943-s001.zip › foods-848626-supplementary.pdf]

Supplementary materials

Sourdough Fermentation Degrades Wheat Alpha-Amylase/Trypsin Inhibitor (ATI) and Reduces Pro-Inflammatory Activity

Xin Huang [1,2,*], Detlef Schuppan [3,4], Luis E. Rojas Tovar [5], Victor F. Zevallos [3,6], Jussi Loponen [2] and Michael Gänzle [5,7]

[1] Department of Food and Nutrition, Faculty of Agriculture and Forestry, University of Helsinki, PL 66, FI-00014 Helsinki, Finland
[2] Fazer Lab, Fazer Group, 01230 Vantaa, Finland; jussi.loponen@fazer.com
[3] Institute of Translational Immunology and Research Center for Immunotherapy, University Medical Center, Johannes Gutenberg University, 55131 Mainz, Germany; detlef.schuppan@unimedizin-mainz.de (D.S.); victor.zevallos@northumbria.ac.uk (V.F.Z.)
[4] Division of Gastroenterology, Beth Israel Deaconess Medical Center, Harvard Medical School, Boston, MA 02215, USA
[5] Department of Agricultural, Food and Nutritional Science, University of Alberta, Edmonton, AB T6G 2P5, Canada; rojastov@ualberta.ca (L.E.R.T.); mgaenzle@ualberta.ca (M.G.)
[6] Nutrition and Food Research Group, Department of Applied and Health Sciences, University of Northumbria, Newcastle Upon Tyne NE1 8ST, UK
[7] College of Bioengineering and Food Science, Hubei University of Technology, Wuhan 430068, China
* Correspondence: xin.huang@helsinki.fi.

Band 1

>sp|P17314|IAAC3_WHEAT Alpha-amylase/trypsin inhibitor CM3 OS=Triticum aestivum OX=4565 PE=1 SV=1

1 MACKSSCSLLLLAAVLLSVLAAASASGSCVPGVAFRTNLLPHCRDYVLQQ

51 TCGTFTPGSKLPEWMTSASIYSPGKPYLAKLYCCQELAEISQQCRCEALR

101 YFIALPVPSQPVDPR SGNVGESGLIDLPGCPR EMQWDFVR LLVAPGQCNL

151 ATIHNVR YCPAVEQPLWI

Band 2

>sp|P16851|IAAC2_WHEAT Alpha-amylase/trypsin inhibitor CM2 OS=Triticum aestivum OX=4565 PE=1 SV=2

1 MASKSSITHLLLAAVLVSVFAAAAATGPYCYPGMGLPSNPLEGCR EYVAQ

51 QTCGVGIVGSPVSTEPGNTPR DRCCKELYDASQHCRCEAVRYFIGRTSDP

101 NSGVLKDLPGCPREPQRDFAKVLVTPGHCNVMTVHNTPYCLGLDI

Figure S1. Protein sequences of identified ATI tetramers. Unique peptides underlined were identified by MALDI-TOF/TOF.

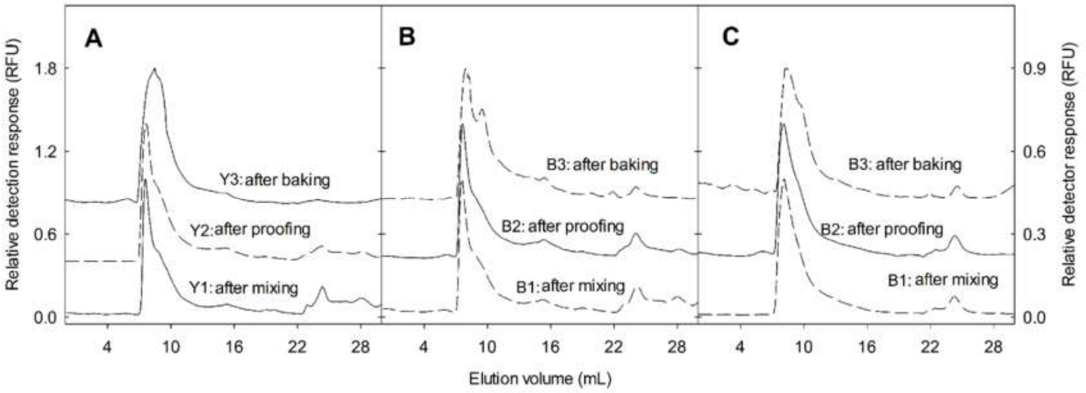

Figure S2. Size-exclusion chromatogram of fluorescence-labelled ATI in bread-making, **Panel A**: Yeast-fermented bread, **Panel B**: (III) chemically acidified dough at pH 5.0, **Panel C**: (V) Rye starter-wheat sourdough. FITC-labelled ATI was added to bread dough. ATI was extracted after mixing of sourdough, after sourdough fermentation, or after mixing of bread dough, after proofing, and after baking. FITC-ATI was separated by size exclusion chromatography coupled to a fluorescent detector set at excitation 488 nm and emission 530 nm. Chromatograms were normalized to the highest peak intensity in each chromatogram, and were offset by 0.4 RFU. The elution volume of the molecular marker bovine serum albumin (66 kDa) was 7.25 mL, of lysozyme (14 kDa) was 9.86 mL, and of glutathione (307 Da) was 17.35 mL. The Roman number and the sample letters are the same as those indicated in Table 1.
